# Calafate (*Berberis buxifolia* Lam.) Berry as a Source of Bioactive Compounds with Potential Health-Promoting Effects: A Critical Review

**DOI:** 10.3390/antiox14111272

**Published:** 2025-10-22

**Authors:** Jaime Ortiz-Viedma, Claudia Vergara, Tamar Toledo, Liliana Zura-Bravo, Marcos Flores, Constanza Barrera, Roberto Lemus-Mondaca

**Affiliations:** 1 Department of Food Science and Chemical Technology, Faculty of Chemical Sciences and Pharmaceutical, Universidad de Chile, St. Dr. Carlos Lorca 964, Independencia, Santiago 8330601, Chile; jaortiz@uchile.cl (J.O.-V.); constanza.barval@gmail.com (C.B.); 2 Department of Basic Sciences, Faculty of Sciences, Santo Tomas University, Avenida Carlos Schorr 255, Talca 3460000, Chile; claudiavergarava@santotomas.cl; 3 Department of Analytical Chemistry, Nutrition and Food Science, Food Technology Division, School of Veterinary Sciences, University of Santiago de Compostela, 27002 Lugo, Spain; tamar@ug.uchile.cl; 4 Food Science Lab, Faculty of Medicine and Health Sciences, Universidad Central de Chile, Santiago 8330546, Chile; 5 Department of Horticulture, Faculty of Agricultural Sciences, University of Talca, Av. Lircay s/n, Talca 3460000, Chile; marcos.flores@utalca.cl

**Keywords:** calafate, production, anthocyanins, antioxidants, healthy

## Abstract

Calafate berry, an ancient perennial shrub of South America (Chile and Argentina), produces a high antioxidant capacity berry with a high polyphenol (1344.2–6553 mg GAE/100 g d.w.) and anthocyanin (26.5–80 mg C-3-G/100 g d.w.) content. The beneficial effects of calafate berries on human health are related to the anti-inflammatory, hypoglycemic, anticancer, and antioxidant properties that the berries possess, which have been confirmed through evidence to date, primarily from in vitro, ex vivo, and animal studies. Several investigations have shown a relationship between the consumption of calafate and a reduction in the risk of contracting cardiovascular diseases (CVD). This was evident in changes in plasma level biomarkers related to CVD, such as thrombomodulin (−24%), adiponectin (+68%), sE-selectin (−34%), sICAM-1 (−24%) and proMMP-9 (−31%), and changes in the production of OH radicals in plasma (−17%) after calafate intake. Calafate may have an antithrombotic role that supports cardiovascular health by lowering the Atherogenic and Cardiovascular Risk Indices. Various authors indicate delphinidin-3-glucoside (384–386 mg/100 g) as the primary bioactive compound responsible for the beneficial properties of Calafate. Although some studies report calafate’s health benefits, scientific evidence, especially in humans, remains limited. Meanwhile, Chile is working to domesticate and cultivate calafate, aiming to turn it from a wild native berry into a sustainable crop for use in the antioxidants and nutraceuticals industry. The lack of human clinical trials emphasizes the need for future research to validate calafate’s health benefits berry.

## 1. Introduction

Berries are recognized as dietary sources of bioactive phytochemicals, with phenolic constituents, especially anthocyanins and other flavonoids [1], extensively linked to antioxidant, anti-inflammatory, and other health-promoting activities. These compounds often underpin both organoleptic properties and nutraceutical potential attributes [2].

Calafate (*Berberis buxifolia* Lam. or *Berberis microphylla*), also known as Magellan barberry, is a wild berry with an intense blue-black color that is harvested in Southern Argentina and Chile. The pre-Columbian people of Patagonia in South America used the calafate fruit to prepare drinks and remedies due to its intense flavor and high potential as a dye. A decoction of the bark brings down a fever, whereas fruits are used to combat diarrhea [3]. Some foliar extracts demonstrated an excellent sun protection factor [4]. Additionally, these extracts have been reported to possess astringent, antipyretic, analgesic, antibacterial, and antiviral activities [5,6]. Multiple studies have confirmed the berries’ properties, including antioxidant, hypoglycemic, anti-inflammatory, anticancer, antimicrobial, and vasodilator activities [7].

Phytochemical profiling of calafate consistently indicates a phenolic-rich matrix dominated by anthocyanins and other flavonoids [8]. Targeted analyses have identified delphinidin and its glycosides (e.g., delphinidin-3-glycosides), petunidin and malvidin derivatives among the major anthocyanins reported in calafate, and total phenolic concentrations that are high in comparison with many temperate berries [9].

This review, therefore, aims to synthesize current knowledge on Calafate berry with an explicit focus on general features of calafate berries, the composition and quantitation of phenolic compounds (including individual anthocyanin profiles), the linkage between these compounds and reported in vitro/in vivo bioactivities, and the gaps and uncertainties regarding their potential use as a source of bioactive compounds with potential health-promoting effects. This manuscript aims to provide a balanced perspective that supports both future biochemical research and the responsible valorization of Calafate berry as a functional food ingredient.

## 2. General Aspects of Calafate Berry

On the other hand, publications on calafate are rare in scientific literature, possibly due to the limited growth zone of the calafate berry in Chile and Argentina. Hence, materials for investigation are not readily available, as in the case of blueberries or murta berries. National institutions in Chile, such as the Instituto de Investigaciones Agropecuarias (INIA), are promoting the domestication of calafate berries to showcase them as a raw material for the antioxidant industry, thereby valorizing their potential as a rich source of phenolic compounds with excellent antioxidant properties [10].

### 2.1. Taxonomy and Botanical Aspects

**Figure 1:**
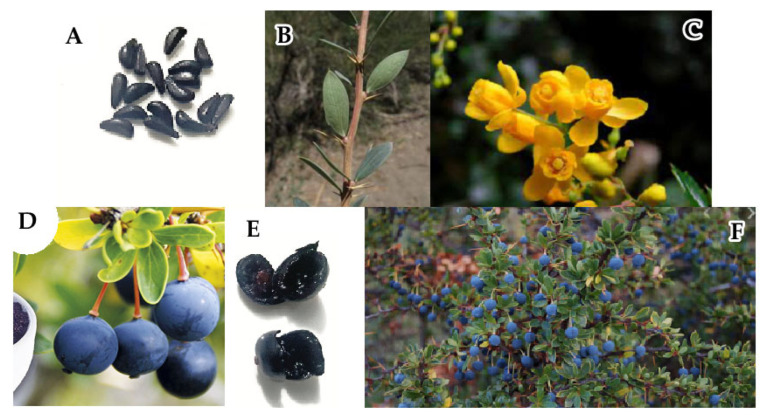
(**A**): Calafate seeds [13]; (**B**): Calafate steam; (**C**): Calafate flowers; (**D**): Calafate berry; (**E**): Calafate pulp [13]; (**F**): Calafate bush.

The berries of calafate (*Berberis buxifolia* Lam.), as shown in Figure 1, are edible, blackish blue, with a diameter of 4–6 mm [11], and have 6 to 10 brown or black seeds. They are appreciated for their high content of polyphenols and antioxidants [11,12]. The fruit is harvested individually from the bush, and due to the spiny nature of the bushes, the cost of commercialization is high, and therefore, products with calafate are expensive.

### 2.2. Distribution, Habitat, and Cultivation Features

The Andes mountains, which extend through Patagonia, largely determine the region’s environmental conditions and cause a climatic contrast between the eastern and western sides. Climatic characteristics influence the structure and functioning of ecosystems [11]. In Chilean and Argentinian Patagonia, the genus Berberis is represented by 16 species of native shrubs, among which *Berberis microphylla* stands out due to its abundance in the region [14,15].

The berries are harvested from January to March, but this period depends on the geographical area where the collection is carried out [16]. Fruiting performance is related to the environmental physiology in which the shrubs develop. Calafate (*Berberis microphylla*) remains predominantly wild-harvested in Chilean and Argentine Patagonia, and official national production statistics are scarce or not systematically reported [17]. Recent population-genetic work underscores its domestication potential while confirming that current supply relies primarily on wild populations [18].

### 2.3. Traditional Use

**Figure 2:**
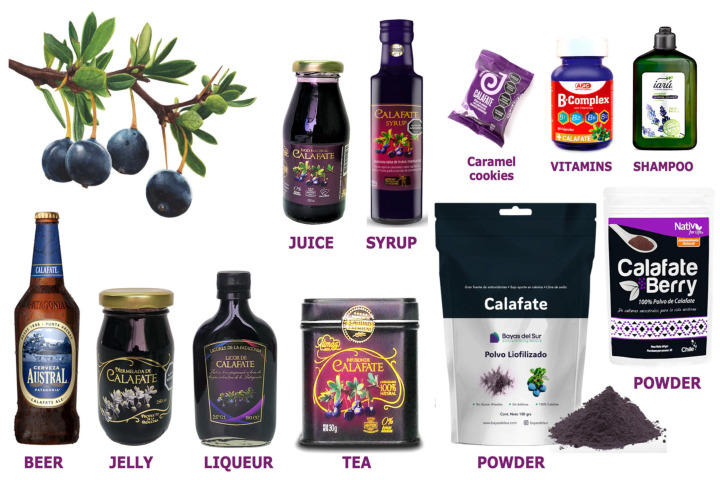
Products made from calafate sold in Argentina and Chile [22,23,24,25,26].

Calafate (*Berberis buxifolia* Lam.) is traditionally eaten fresh, especially in cooking; however, due to its high water content and soft texture, it is very perishable and often processed into shelf-stable foods such as teas, jams, ice creams, candies, and liqueurs, as well as nutraceutical products like freeze-dried powders and capsules (Figure 2). It is also used in non-food applications such as cosmetics, although many of these remain artisanal and locally distributed [16]. Beyond these uses, calafate has increasingly entered niche markets through value-added products (Figure 2), such as regional beers, liqueurs, and commercial powders. This trend aligns with advances in scientific and technological research on its processing and stabilization, including cryo/freezing-based concentration methods [19], encapsulation for functional formulations (Romero-Román et al., 2021) [20], and cryo-concentration combined with high-pressure homogenization to improve extract quality and safety [5]. Overall, these developments underscore the cultural and commercial significance of calafate, while underscoring the need for further research to support its sustainable use, promote market growth, and establish scientific validation. Mainly in local markets in Southern Chile and Argentina [21] (Instituto Interamericano de Cooperación para la Agricultura, 2018).

## 3. Nutritional Composition of Calafate Berry

The unidirectionality in most of the studies of calafate is striking. Most scientific studies focused on the antioxidant activity, measurement, and identification of bioactive compounds, providing a general overview of the fruit’s characteristics. This demonstrates the authors’ commitment to thoroughly investigating and clarifying this topic. However, the characterization of the raw material is an issue that needs to be addressed, as it downplays the importance of conducting a proximate analysis of the fruit and ignores the valuable information that can be obtained. That information is reflected in Table 1, which includes only four studies that provided nutritional information in their results.

One of the reasons that could cause the scarce analysis of the fruit’s proximate composition is the shrub’s limited growth region. The geographical location where the calafate berries thrive is the Patagonia region of Argentina and Chile. As a result, it is not a widespread shrub and fruit known worldwide, unlike other berries such as raspberries or blueberries, which are produced in larger quantities and exported to multiple countries. In the case of calafate, the harvest volumes need to be increased to meet global export demand. The exploitation is irregular, lacking a formally established offer, and the harvest is conducted by occasional collectors between January and February, resulting in significant differences in the volumes offered each season.

As shown in Table 1, Pino et al. (2018) [13] and Ruiz et al. (2010) [17] determined similar moisture contents in the pulp of Chilean calafate, which is significantly lower than that found in the Argentine samples [11,27], indicating substantial quality differences. The Argentine varieties showed significantly lower carbohydrate and protein but higher fat and mineral content. The high moisture content of the calafate berries will likely impact their shelf-life and make them susceptible to water loss or deterioration in organoleptic quality.

### 3.1. Carbohydrates

The content of soluble sugars has been revealed in ecological and agronomic studies to correlate strongly with stress tolerance [27]. The accumulation of carbohydrates also limits fungal colonization and controls osmotic potential in plant cells [11]. Sugars are the primary nutrients found in the fruit pulp [13] and contribute to the quality of the fruit, such as weight, firmness, color, and flavor [11]. As shown in Table 1, there are differences in the carbohydrate values of Chilean and Argentine calafate. The harvesting area could explain these differences, as could the climatic conditions and the degree of maturity of the analyzed fruit [28]. On the other hand, the glucose and fructose content of calafate berries has been reported to range from 0.3 to 5% of fresh weight [17]. Regarding soluble solids, Pino et al. (2018) [13] reported a value of 26.4 °Brix and described a wider range of variability, from 8 to 20 °Brix.

### 3.2. Proteins

In terms of protein composition, analyses of Argentine calafate berries have shown that proteins are concentrated primarily in the seeds, where levels are approximately tenfold higher than in the pulp. Calafate fruits have therefore been proposed as a potential source of protein [11]. Nevertheless, when expressed on a fresh weight basis, the protein content of *Berberis buxifolia* Lam. is markedly lower—by a factor of three to four—than that reported for other Berberis species, such as *Berberis vulgaris* and *Berberis pimana* [17].

### 3.3. Vitamin C

In addition, calafate berries are a rich source of vitamin C, with concentrations ranging from 41.2 to 117.5 mg ascorbic acid (AA) per 100 g of fruit [29], which significantly contributes to their overall antioxidant capacity [17]. Remarkably, the ascorbic acid content of calafate far exceeds that of other widely consumed berries, such as blueberries (4–8 mg AA/100 g), blackberries (6–19 mg AA/100 g), and raspberries (18–40 mg AA/100 g) [30].

### 3.4. Lipids

Regarding lipid composition, calafate pulp contains only trace amounts of fat, a trait that markedly reduces the likelihood of lipid oxidation. Regarding lipid composition, calafate pulp contains only trace amounts of fat, a trait that markedly reduces the possibility of lipid oxidation. The lipid content of calafate seeds is substantially higher than that of the pulp, reaching levels comparable to those reported for avocado pulp (15.39%) [31].

### 3.5. Fatty Acids

Table 2 presents values reported in the literature on the fatty acid profile of oils obtained from pomace, seeds, and fresh fruit of berries, particularly calafate and maqui. Although the analytical methodologies differed, the results were expressed as percentages, except for some data from fresh calafate fruit, which were described in mg/g of fresh weight. According to the collected data, oils derived from the pomace, seed, or fresh fruit of calafate present a lipid profile predominantly composed of palmitic (16:0), oleic (18:1), linoleic (18:2), and α-linolenic (18:3) fatty acids. This profile is comparable to that of other berries, such as maqui, strawberry, blackberry, and cranberry, as shown in Table 2. In the case of calafate pomace oil, the predominant presence of α-linolenic acid (ALA) (36.7–37.9%) stands out, followed by linoleic acid (LA) (30–31.8%) and oleic acid (19.2–22.2%). This positions this oil as a potential functional ingredient, as linoleic and α-linolenic acids are metabolic precursors of long-chain polyunsaturated fatty acids (LCPUFAs). Specifically, LA is a precursor of arachidonic acid (C20:4 n-6, AA), which plays a key role in immune system activity and brain development [32,33]. On the other hand, ALA is a precursor of eicosapentaenoic acid (C20:5 n-3, EPA) and docosahexaenoic acid (C22:6 n-3, DHA).

Various studies suggest that consuming unsaturated fatty acids positively affects cardiovascular health by reducing triglycerides, inflammatory markers, and the levels of reactive oxygen species (ROS) in cells exposed to high glucose levels [32,33,34,35]. In contrast, Olivares-Caro et al. (2020) [2] reported the fatty acid profile of oil extracted from fresh calafate fruit, where saturated fatty acids predominate, particularly myristic acid (0.16 mg/g fresh weight), followed by palmitic acid (0.08 mg/g fresh weight) and stearic acid (0.06 mg/g fresh weight). However, as in pomace, the predominant unsaturated fatty acids are linolenic acid (0.09 mg/g fresh weight), followed by linoleic acid (0.07 mg/g fresh weight) and oleic acid (0.04 mg/g fresh weight). These results suggest this oil may have greater stability due to its higher saturated fatty acid content [36].

The FAO recommends a high polyunsaturated/saturated fatty acids (PUFA/SFA) ratio in the diet, as there is evidence that a diet rich in PUFA and low in SFA reduces the risk of fatal coronary diseases [37]. On the other hand, unlike calafate, maqui presents a similar lipid profile in the seed, fruit, and pomace, with a higher content of unsaturated fatty acids. Linoleic acid (44.9–46%) and oleic acid (32–39%) are notable, along with a significant percentage of saturated fatty acids (12–15%). In addition, the palmitic acid (8.5–9.7%) and stearic acid (2.6–3.5%) are the most representative. The use of pomace for oil extraction not only represents an alternative for utilizing by-products, but also offers a suitable lipid profile for industrial, food, or cosmetic applications.

**Table 2:** Fatty acids from different berry species, including Calafate berry.

Berry		Calafate			Maqui			Cranberry	Blackberry	Strawberry
References		[38]	[39]	[2]	[40]	[41]		[42]	[43]	
Location		Magallanes Region	Aysén Region	Punta Arenas	Ñuble Region	Aysén Region	Aysén Region	State of Washington	Panevėžys	
Country		Chile	Chile	Chile	Chile	Chile	Chile	USA	Lithuania	
Fatty acids		Pomace	Pomace	Fresh fruit	Seeds	Fresh fruit	Pomace	Seeds	Pomace	
		%	%	mg/g	%	% *w*/*w*	% *w*/*w*	%	%	%
C12:0	Lauric acid	-	-	-	0.23 ± 0.03	0.51 ± 0.02	0.48 ± 0.01	0.14	-	-
C14:0	Myristic acid	-	-	0.16 ± 0.01	0.65 ± 0.02	1.07 ± 0.04	1.04 ± 0.00	0.08	-	-
C15:0	Pentadecanoic acid	-	-	-	-	0.02 ± 0.00	0.03 ± 0.01	-	-	-
C16:0	Palmitic acid	7.93	8.1 ± 0.8	0.08 ± 0.02	8.55 ± 0.01	8.63 ± 0.11	9.68 ± 0.02	5.38	4.56 ± 0.03	3.66 ± 0.46
C16:1	Palmitoleic acid	-	-	-	0.26 ± 0.01	0.35 ± 0.01	0.50 ± 0.01	-	0.07 ± 0.00	0.14 ± 0.02
C18:0	Stearic acid	2.58	2.7 ± 0.1	0.06 ± 0.00	2.63 ± 0.05	3.47 ± 0.09	3.46 ± 0.04	1.25	3.76 ± 0.06	1.28 ± 0.17
C18:1	Oleic acid	19.18	22.2 ± 0.7	0.04 ± 0.01	39.71 ± 0.04	33.49 ± 0.11	32.26 ± 0.09	25.30	21.14 ± 0.36	12.91 ± 1.51
C18:1	Vaccenic acid	0.61	-	-	-	3.81 ± 0.01	3.5 ± 0.05	-	-	-
C18:2	Linoleic acid	31.79	30.0 ± 0.1	0.07 ± 0.03	45.81 ± 0.05	46.00 ± 0.35	44.89 ± 0.33	37.68	59.44 ± 0.93	40.67 ± 4.81
C18:3	Linolenic acid	37.9	36.7 ± 0.2	0.09 ± 0.04	2.35 ± 0.02	1.96 ± 0.04	3.39 ± 0.21	30.09	9.14 ± 0.15	33.90 ± 3.98
C20:1	Eicosenoic acid	-	-	-	-	0.24 ± 0.01	0.22 ± 0.00	-	0.55 ± 0.13	0.60 ± 0.09
C20:4	Arachidonic acid	-	-	-	-	-	-	0.07	-	-
C22:0	Behenic acid	-	-	-	-	0.18 ± 0.01	0.27 ± 0.02	-	-	0.13 ± 0.02
C22:1	Erucic acid	-	-	0.08 ± 0.01	-	-	-	-	-	-
C24:0	Lignoceric acid	-	-	-	-	0.13 ± 0.01	0.11 ± 0.13	-	-	-
SFA		10.52	10.8	0.3	12.06	14.01	15.07	6.92	8.32	5.07
MUFA		19.18	22.2	0.12	39.97	37.89	36.48	25.14	21.76	13.65
PUFA		70.31	66.7	0.16	48.16	47.96	48.28	67.98	68.58	74.57
PUFA/SFA ratio		6.69	6.18	0.53	3.99	3.42	3.20	9.88	8.24	14.71

### 3.6. Tocopherols

Tocopherols and tocotrienols fall within the category of fat-soluble vitamins (vitamin E), essential for normal human growth, as the human body cannot synthesize them and must be obtained through food or supplements [44,45,46,47]. According to Kamal-Eldin et al. (2015) [48], the antioxidant mechanism of tocopherols in lipids is based on their ability to donate a proton to lipid peroxyl radicals (LOO^•^), preventing the propagation of lipid peroxidation. Initially, the tocopherol (TOH) and the peroxyl radical come into proximity, forming a charge-transfer transition state, through which proton tunneling occurs from the hydroxyl group of the tocopherol to the radical, generating a lipid hydroperoxide (LOOH) and a chromanoxyl radical (TO^•^), which is stabilized by electron delocalization [48].

Alpha-tocopherol is the most biologically active and has been associated with the ability to prevent lipid peroxidation in membranes and low-density lipoproteins [49]. Thanks to their antioxidant activity, tocopherols have generated significant interest due to their impact on oxidative stability, nutritional quality, and shelf life of oils and other products. Table 3 presents the content of tocopherols and tocotrienols in oils extracted from the seeds, pomace, or fruit of calafate and maqui, as well as other berries, including blackcurrant, blueberry, and cranberry. It can be observed that all berries have a diverse composition of tocopherols and tocotrienols, with α-tocopherol and γ-tocopherol being the predominant forms, as well as α-tocotrienol and γ-tocotrienol. In particular, the high content of α-tocopherol in the fruit and pomace oil of maqui (735.0 and 444.2 ppm, respectively) stands out, in contrast to the results found for calafate, which, although lower, are still significant, with 112.1 ppm for α-tocopherol and 116.84 ppm for β-tocotrienol. This indicates a complementary antioxidant profile, which may suggest better antioxidant capacity. Previous studies have shown that tocopherol mixtures exhibit superior antioxidant activity compared to isolated α-tocopherol, as γ- and δ-tocopherols possess anti-inflammatory properties and can counteract the pro-oxidant effect of α-tocopherol [50]. Moreover, the stability of tocopherol-rich oils can be enhanced by synergistic compounds such as polyphenols, phospholipids, and carotenoids [50,51]. Therefore, from an industrial perspective, berry oils such as calafate are promising for various applications, leveraging their high antioxidant content in functional oil formulations, cosmetic and skincare products, and pharmaceutical products targeting cardiovascular protection and modulation of inflammatory processes.

On the other hand, it is worth noting studies on tocopherols and their impact on human health. Although various preclinical studies have highlighted the positive antioxidant and anti-inflammatory properties of tocopherols and tocotrienols, especially in in vitro and animal models [46,52,53], clinical evidence in humans regarding their benefits in the prevention or treatment of chronic diseases such as cardiovascular disease, type 2 diabetes, dementia, certain types of cancer, or eye disorders is limited and, in many cases, contradictory. For example, research has suggested that vitamin E supplementation may delay functional decline in patients with moderate Alzheimer’s disease, although without significant improvements in cognitive function. However, other studies have found no beneficial effects in preventing the progression from mild cognitive impairment to dementia [54,55,56,57]. Regarding cancer, most clinical trials have not shown a significant reduction in the incidence of various types of cancer with vitamin E supplementation; in fact, some studies have reported an increased risk of prostate and lung cancer with high doses, despite other studies showing positive results of α-tocopherol supplementation in prostate cancer prevention.

Regarding eye diseases such as age-related macular degeneration, results are also inconsistent, with no conclusive evidence of benefits [47,58,59,60,61,62,63,64,65]. These discrepancies may be attributed to factors such as individual genetic variability, the different forms and doses of vitamin E used in the studies, and the complexity of the diseases evaluated. Therefore, although the antioxidant mechanisms of vitamin E are well recognized, its clinical efficacy in humans for preventing or treating chronic diseases still requires more robust and conclusive research.

**Table 3:** Tocopherols from different berry species, including Calafate berry.

Berry	Calafate		Maqui				Cranberry	Blueberry	Blackcurrant
References	[38]	[39]	[66]	[40]	[67]	[38]	[68]	[42]	[69]
Location	Magallanes Region	Aysén Region	Bio-Bio Region	Ñuble Region	Lanco	Magallanes Region		State of Washington	Rijkevorsel
Country	Chile	Chile	Chile	Chile	Chile	Chile	EC *	USA	Belgium
Compound	Pomace (oil)	Pomace (oil)	Dry fruit	Seeds (oil)	Dry fruit (oil)	Pomace (oil)	Press residues	Seed (oil)	Seed (oil)
	ppm	ppm	ppm	ppm	ppm	ppm	ppm	ppm	ppm
α-tocopherol	112.10 ± 0.11	75.4 ± 3.8	4.5 ± 0.1	169.33 ± 11.39	735 ± 19	444.20 ± 0.13	8.7	4.4 ± 0.2	335.1
β-tocopherol	11.80 ± 0.14	-	-	7.78 ± 2.34	5 ± 1	14.79 ± 0.20	1.6	-	34.5
γ-tocopherol	3.03 ± 0.12	-	-	56.76 ± 2.98	97 ± 4	18.98 ± 0.09	1.5	34.4 ± 0.1	12.2
δ-tocopherol	4.28 ± 0.05	-	-	13.58 ± 3.5	7 ± 1	1.14 ± 0.05	-	-	239.9
α-tocotrienol	67.31 ± 0.05	127.7 ± 4.0	-	323.80 ± 20.3	53 ± 0	46.31 ± 0.05	10.1	-	-
β-tocotrienol	116.84 ± 0.1	-	-	20.20 ± 5.99	-	27.43 ± 0.10	-	-	-
γ-tocotrienol	13.84 ± 0.12	203.7 ± 10.5	-	5.74 ± 1.05	-	23.02 ± 0.15	39.2	330.4 ± 11.4	-
δ-tocotrienol	-	-	-	53.9 ± 7.42	-	-	-	6.0 ± 1.0	-
Total tocols	329.20 ± 0.11	406.4	-	327.3 ± 19.32	897	575.86 ± 0.12	61.1	375.2 ± 12.4	621.7

* EC: European Community.

## 4. Phenolic Compounds in Calafate Berry

Calafate berries are rich in anthocyanins and polyphenols [12]. The high content of anthocyanins is responsible for the color of the fruits. It represents an excellent natural pigment source increasingly used in juices and other beverages to replace synthetic colorants [10]. Anthocyanins are not solely natural colorants; they are bioactive compounds with high antioxidant activity. Therefore, consuming calafate in the diet has been associated with beneficial effects on human health. The chemical characteristics and antioxidant capacity of calafate and other Chilean native fruits depend on the genetic differences between the species [70], the climatic conditions, the growth location of the plant [28], as well as on the state of maturity of the fruit at harvest, so that these variations will affect the antioxidant capacity of the fruits [28,71].

Studies have shown calafate to be a rich source of phenolic antioxidants [72]. The main phenolic compounds present in the calafate berries are gallic acid, chlorogenic acid, and rutin [28]. Some reports have shown that antioxidant activity has a weak correlation with anthocyanins but a higher correlation with phenolic compounds, suggesting that other aromatic compounds likely contribute to the total antioxidant activity of calafate berries [72]. Polyphenols are bioactive compounds that stand out for their ability to capture free radicals, thereby acting as natural antioxidants. Thus, a higher polyphenol content is associated with higher antioxidant activity in the fruit.

### 4.1. Polyphenols

**Figure 3:**
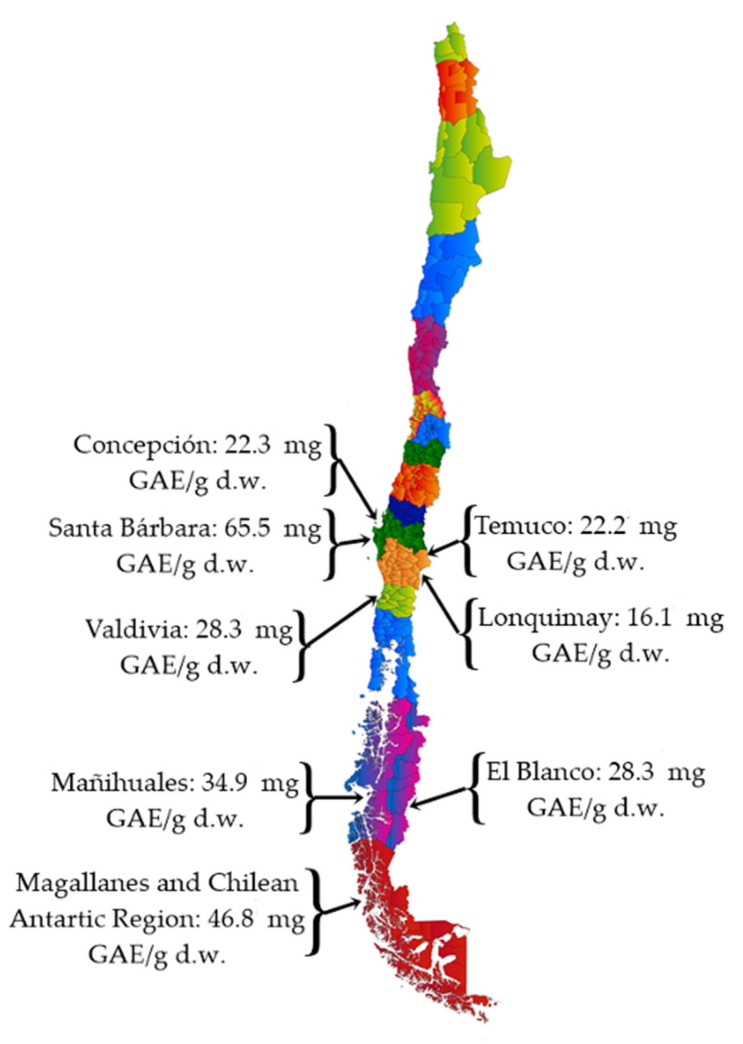
Polyphenol content from calafate berries in different locations in Chile [11,13,28,70,74,75].

As shown in Table 4, the content of polyphenols measured using the Folin–Ciocalteu method varies between 1344.2 ± 10.50 and 6553 ± 1.35 mg GAE/100 g d.w. (dry weight) in extracts of calafate berries, which are comparable to the native Chilean maqui berries that have a content between 1906.5 ± 73.20 and 5944.9 ± 793.80 mg GAE/100 g d.w. [73,74]. On the other hand, blueberry has a range between 458 ± 3.46 to 1229.6 ± 20.90 mg GAE/100 g d.w. [70,74]. The anthocyanin content is usually determined by the pH-differential method and expressed as cyanidin-3-glucoside (C-3-G). In calafate berries, anthocyanin content varies between 26.5 ± 0.01 and 66 ± 0.30 mg C-3-G/100 g d.w., while in maqui berries and blueberries, values of 72.70 ± 0.10 mg C-3-G/100 g d.w. [74], and 20.10 ± 1.65 mg C-3-G/100 g d.w. [70,74] have been reported. This indicates that the calafate berries contain the same level of anthocyanin as familiar groups of berries. Figure 3 shows the polyphenol content in calafate berries in different locations from where fruit samples were collected for the respective studies. As can be seen, the polyphenol content varies according to the city/region, depending on factors such as harvest date, locality, environmental physiology, and solar radiation, among others. The berries collected in Santa Bárbara had the highest content of polyphenols, at 65.53 mg GAE/g dry weight (d.w.), followed by the Magallanes and the Chilean Antarctic Region, with polyphenol contents of 46.8 mg GAE/g d.w. It is expected that the fruits of these areas will have an antioxidant power directly related to their polyphenol content.

### 4.2. Antioxidant Capacity

Different methods can be used to determine antioxidant activity, although all techniques are based on the same principle related to the entrapment of free radicals. The antioxidant activity was determined by FRAP (Ferric Ion Reducing Antioxidant Power) assays, resulting in a value of 38.9 ± 1.70 mmol Fe^2+^/100 g d.w. for blueberries and 5.9 ± 0.10 mmol Fe^2+^/100 g d.w. for blueberries [74]. Different FRAP values have been reported for the calafate berry, specifically 11.7 ± 1.8 mmol Fe^+2^/100 g d.w. [74] and 38.44 ± 0.08 mmol Fe^2+^/100 g d.w. [75]. The FRAP assay measures the reduction of Fe^3+^ ions to Fe^2+^ ions, indicating that the antioxidant power of the sample is directly proportional to the amount of Fe^+2^ formed. Consequently, calafate and maqui may be considered to have similar antioxidant power that would be superior to that of blueberries. The antioxidant activity of calafate berries has also been determined by DPPH-IC_50_ assays, which measure the sample size required to reduce the DPPH free radicals by 50%. In this case, a smaller sample would indicate higher antioxidant capacity. As shown in Table 4, extracts of calafate berries exhibit IC_50_ values ranging from 2.33 ± 0.21 to 12.6 ± 0.38 μg/mL; this variation is likely due to the different locations from which the fruit samples were collected. In comparison, Ramirez et al. (2015) [70] reported an IC_50_ value of 3.32 ± 0.18 μg/mL for blueberries, while Rodoni et al. (2014) [72] reported that calafate (*Berberis buxifolia* Lam.) berries have a radical scavenging capacity of ten times greater than that of apple, orange, or pear. According to Table 4, Ortiz-Viedma et al. (2022) [39] conducted a biorefinery process using supercritical CO_2,_ followed by a pressurized ethanol/water mixture, on freeze-dried calafate berry pomace. The biorefinery process extracted high levels of total polyphenol, anthocyanin content, and antioxidant compounds (by ORAC and DPPH) comparable to those obtained in pressed pomace from other berries.

### 4.3. Anthocyanin

**Figure 4:**
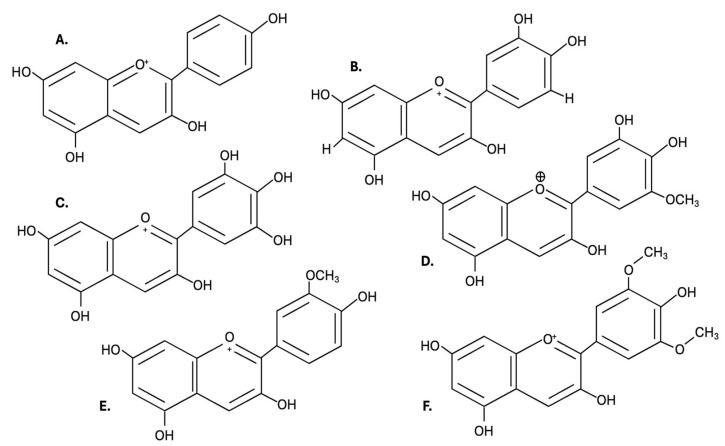
Structure anthocyanidins: (**A**): Pelargonidin; (**B**): Cyanidin; (**C**): Delphinidin; (**D**): Petunidin; (**E**): Peonidin; (**F**): Malvidin.

More than 600 anthocyanins derived from six anthocyanidins (cyanidin, peonidin, petunidin, malvidin, pelargonidin, and delphinidin) have been found in edible plants. However, studies have demonstrated the predominance of only 23 anthocyanidins in nature [1]. Anthocyanins are composed of an anthocyanidin molecule with at least one attached glycoside. The anthocyanins are subdivided into sugar-free anthocyanidin aglycones and anthocyanin glycosides. Anthocyanidins share a basic structure, and the anthocyanidin that will originate depends on the substitution in the R1 and R2 positions (Figure 4).

**Figure 5:**
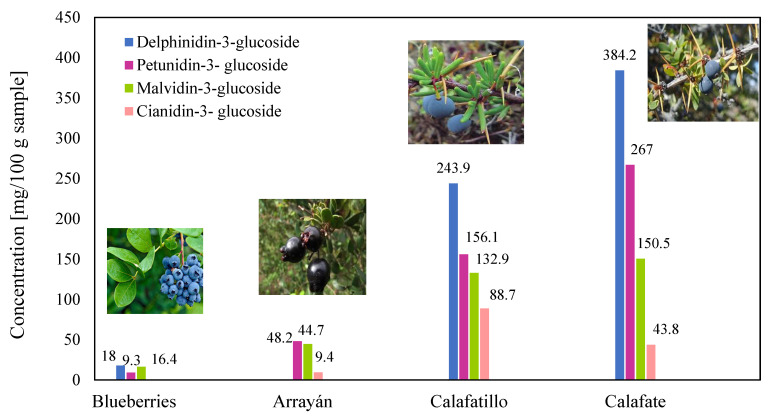
Anthocyanins in Calafate, Blueberries, Arrayán and Calafatillo [2,7,9,27,30,39,70,71,74,76,77,79,80,81].

**Figure 6:**
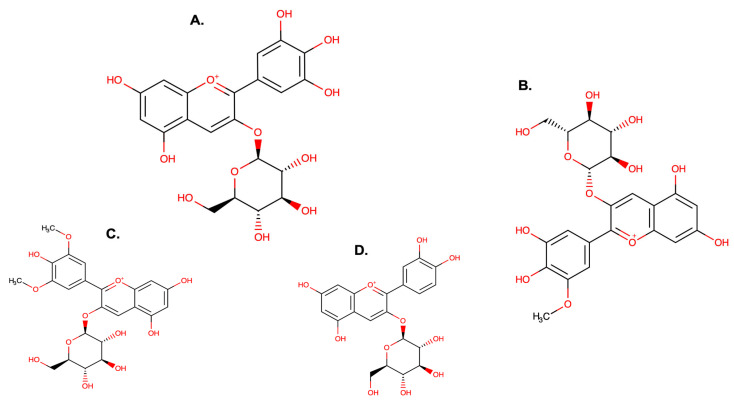
Structures of specific anthocyanins present in calafate: (**A**). Delphinidin-3-glucoside; (**B**). Petunidin-3-glucoside; (**C**). Malvidin-3-glucoside; and (**D**). Cyanidin-3-glucoside.

Cyanidin and peonidin are responsible for plants’ red and orange colors, while malvidin, delphinidin, and petunidin produce red and blue colors. Therefore, the levels of anthocyanins in the fruits are responsible for the difference in color intensity [70], which further contributes to variations in antioxidant activity [1]. The structure and color of anthocyanins change reversibly with pH. Furthermore, stress conditions such as light, temperature, oxygen, and pH cause the degradation of anthocyanins. Fourteen different anthocyanins have been found in calafate, most of which are glycosylated derivatives of delphinidin, petunidin, peonidin, cyanidin, and malvidin, as shown in Figure 5 [70]. Calderón-Reyes et al. (2020) [77] suggest that the diversity of anthocyanins present in calafate berries is the responsible factor for the high antioxidant capacity, while Lazze et al. (2004) [78] indicate that delphinidin would be responsible for the antioxidant capacity due to its chemical structure. Lazze et al. (2004) [78] studied the effect of the anthocyanins delphinidin and cyanidin on cancer cells, concluding that delphinidin exhibits more effective biological activity than cyanidin due to its hydroxyl groups in its ring. However, the antioxidant properties of anthocyanins shown in in vitro studies are not necessarily evidence for the antioxidant effects in humans after consuming foods rich in anthocyanins. Also, the structures of specific anthocyanins present in calafate (delphinidin, petunidin, peonidin, cyanidin, and malvidin) are shown in Figure 6.

Table 4 shows the main anthocyanins present in calafate berries and various other berries, showing that delphinidin-3-glucoside, petunidin-3-glucoside, and malvidin-3-glucoside predominate in calafate berries. Calderón-Reyes et al. (2020) [77] found that the anthocyanin present in the highest proportion in calafate extracts was delphinidin, followed by malvidin, petunidin, cyanidin, and finally, peonidin, with low concentrations. This finding aligns with those of Ruiz et al. (2013) [30] and Olivares-Caro et al. (2023) [7], who observed a predominance of anthocyanins based on delphinidin and petunidin, with delphinidin-3-glucoside being the most abundant compound. On the other hand, Brito et al. (2014) [9] obtained delphinidin-3-galactoside as the main anthocyanin, followed by petunidin-3-glucoside and malvidin-3-glucoside. Boeri et al. (2020) [27] indicated that 57% of the total anthocyanin content belongs to delphinidin-3-glucoside, the main anthocyanin in the berry. The total anthocyanin levels detected in the Berberis genus were higher than the levels seen in other consumed fruits, such as blueberries, strawberries, blackberries, raspberries, or murtilla [30,72]. The variation in anthocyanin content among berries explains their differing antioxidant capacities [70]. It is worth noting that the antioxidant properties of anthocyanins have been reported in both in vitro and in vivo assays. According to Lazze et al. (2004) [78], some inhibitory effects of anthocyanins on the growth of specific cancer cells have been observed.

## 5. Calafate Berry and Its Health-Promoting Effects

The berries possess anti-tumor and anti-inflammatory properties, which can help prevent insulin resistance and diabetes in humans [13]. The level of antioxidant activity of the fruit of this species exceeds that of other berries of frequent consumption, such as blueberries (*Vaccinium corymbosum*) [11], raspberries (*Rubus idaeus* L.), strawberries (*Fragaria×ananassa*), and blackberries (*Rubus ulmifolius*). Consuming fresh berries and fruit promotes health due to their high content of antioxidant polyphenols, which help protect against cardiovascular diseases. Calafate is considered a superfruit due to its high content of vitamin C and polyphenols such as phenolic acids, flavanols, and anthocyanins. Polyphenol levels are higher than those recorded in other berries, such as blueberries, blackberries, and raspberries. Fruits that have health benefits are called “superfruits”. Nutrition scientists consider superfruits to be “super” when fruits have incredibly high levels of antioxidants, fibers, vitamins, minerals, and other health-enhancing nutrients [82].

Table 5 provides detailed information regarding the type of preparation or extract, the experimental model utilized, the primary biological outcomes observed, and the associated references. This summary enhances the manuscript’s clarity, thereby facilitating a more accurate comparison of the reported effects.

### 5.1. Anti-Inflammatory Effect

Consumption of processed foods induces an inflammatory state in rodents, associated with an intense infiltration of macrophages in adipose tissue. The increase in adipokines (molecules secreted by adipose tissue) is positively correlated with body adiposity, contributing to the generation and maintenance of a chronic inflammatory state associated with obesity [75]. Additionally, it has been recognized that an increase in body adiposity is typically accompanied by an increase in systemic oxidative stress and a condition of low-grade inflammation in adipose tissue [74]. The chronic inflammatory state in adipose tissue contributes to insulin resistance associated with obesity. Evidence suggests that interrupting the inflammatory response caused by obesity allows insulin sensitivity to be restored [75].

Polyphenols have been shown to improve the inflammatory state associated with obesity and enhance insulin sensitivity [87]. Bottini et al. (2007) [84] indicate that calafate leaves and roots have antibiotic properties. However, little is known about the vasodilator properties of the fruit’s components, except for its anti-inflammatory effect and nutritional value, which inhabitants of Patagonia in Southern Argentina utilize. Evidence suggests that interrupting the inflammatory response caused by obesity enables the recovery of insulin sensitivity. This was demonstrated in an in vitro inflammation model, indicating an insulin-sensitizing role for the calafate berry [83]. Nevertheless, this has not been evaluated in vivo. Several inflammatory products, such as nitric oxide (NO), correlate with increased body adiposity and appear to participate in the induction and maintenance of the chronic inflammatory state associated with obesity [74].

Soto-Covasich et al. (2020) [75] reported that the polyphenol-rich calafate extract attenuates the expression of pro-inflammatory markers, contributing to body weight balance by reducing fat deposits. Calafate extract also significantly inhibited the secretion of NO produced by macrophages induced by LPS (lipopolysaccharides), thereby modulating the genetic expression of cytokines associated with inflammatory pathways responsible for NO secretion. The extracts of maqui and cranberry show similar effects; however, the induced impact of calafate extract is more drastic [74].

### 5.2. Hypoglycemic Effect

Type 2 diabetes mellitus is a metabolic disorder characterized by high blood glucose levels resulting from a deficiency in insulin action and secretion. Several species of the genus Berberis are described as having hypoglycemic potential, such as *B. lycium*, *B. aristata*, *B. asiatica*, *B. vulgaris*, *B. integerrima*, *B. ceratophylla*, *B. moranensis* and *B. crataegina* [8]. Furrianca et al. (2017b) [15] studied the effect of the ethanolic extract from the calafate’s root on glucose absorption in a culture of liver cells. They determined a positive correlation between the dose of root extract of *B. microphylla* and glucose uptake. Due to the hypoglycemic effect of the extracts, they represent a potential treatment for type 2 diabetes mellitus. Soto-Covasich et al. (2020) [75] demonstrated the restoration of glucose tolerance in a model of diet-induced obesity, positioning the calafate berry as a natural source of polyphenols that could be used to treat insulin resistance. Reyes-Farias et al. (2016) [83] studied in vitro the glucose absorption capacity of adipocytes when treated with fruit extracts in an insulin-resistant environment. They observed that calafate treatment effectively inhibited glucose uptake by adipocytes.

### 5.3. Antioxidant Activity

Regarding the inhibition of lipid peroxidation, the antioxidant compounds present in fruits play a crucial role in mitigating the spread of diseases related to oxidative stress. Ramirez et al. (2015) [70] used human erythrocytes to perform the lipid peroxidation assay to explore the antioxidant properties of other Chilean berries (blueberry, calafate, murta, myrtle, and chequen). The highest activity was found for blueberries, followed by calafate fruits. These berries prevented hemolysis caused by the rupture of cell membranes induced by lipid peroxidation. The inhibitory activity presented by the calafate extract was close to that of gallic acid and higher than that shown by anthocyanin cyanidin-3-O-glucoside. The extracts of the investigated berries showed that lipid peroxidation inhibitory activity further supports their health benefits and potential therapeutic use [70].

Based on the above, the beneficial effects attributed to calafate (*Berberis buxifolia* Lam.) on human health are strongly linked to its antiradical activity and ability to protect against oxidative stress, as demonstrated mainly in in vitro, ex vivo, and animal models [17]. High antiradical capacity has been consistently observed through standard assays (DPPH, ABTS, FRAP, and ORAC), which closely correlate with the berry’s total phenolic and anthocyanin content [71].

Additionally, it is worth noting that the distribution of bioactive compounds is organ-dependent: pulp and peel concentrate anthocyanins and hydrophilic phenolics, while seeds contain the lipid fraction and tocopherols. This organ specificity suggests that different calafate tissues may play complementary roles in oxidative-stress protection, an aspect that merits further exploration in future research [20].

### 5.4. Antimicrobial Activity

The alkaloid berberine, found in most species of the genus Berberis [11], is responsible for its antimicrobial properties. It is noteworthy that different reports highlight *Berberis buxifolia* Lam. as a significant source of alkaloid compounds. Berberine has also been cited as a toxic agent for insects and vertebrates, and it inhibits the growth of bacteria, fungi, and viruses.

Brito et al. (2014) [9] indicated that the antimicrobial activity of the roots and shoots of calafate (stem and leaves) against Gram-positive bacteria, such as *S. aureus*, *B. cereus*, *S. epidermidis*, and *B. subtilis*, has been associated with the presence of isoquinoline alkaloids, mainly berberine [85]. It is worth noting that the authors primarily used methanol as the solvent for extraction. Ethanol is also commonly used as a solvent. These polar solvents are used to recover polyphenols within a plant matrix. The extraction performance depends on the polarity of the solvent, the chemical composition of the compounds to be extracted, the amount and position of their hydroxyl groups, molecular size, as well as the extraction conditions such as concentration of the solvent, temperature, contact time, particle size, and mass–solvent ratio, among others.

### 5.5. CVD Risk-Reducing Effects Potential

Olivares-Caro et al. (2023) [7] indicated that reducing the risk of cardiovascular disease caused by a high-fat diet, where through the study of mouse plasma metabolome after chronic consumption of calafate extract, changes were evident in plasma biomarkers related to CVD, specifically thrombomodulin (−24%), adiponectin (+68%), soluble endothelial leukocyte adhesion molecule-1 (sE-selectin) (−34%), soluble intercellular adhesion molecule-1 (sICAM-1) (−24%), and pro-matrix metalloproteinase-9 (proMMP-9) (−31%) levels. Furthermore, the production of OH radicals in plasma was reduced (−17%) after calafate intake, where it is believed that these changes could be associated with protection against atherosclerosis [7]. Also, Duarte et al. (2024) [8] acutely administered calafate extract to mice, where the results suggested that calafate extract modulates the expression of thermogenic markers and prevents alterations in the mitochondrial crest and intestinal microbiota in preclinical models; all this provides valuable insights into the potential role of calafate in the treatment of obesity-related metabolic diseases. Lastly, in a study where mice were fed freeze-dried calafate, no significant modifications were observed in dietary variables or total cholesterol and triglyceride concentrations. However, the Atherogenic Index and the Cardiovascular Risk Index were significantly decreased, concluding that calafate could have an antithrombotic function in cardiovascular health [86].

Likewise, Duarte et al. (2024) [8], in their study with mice, obtained results showing no significant differences in the final weight of the mice among the different treatments (high-fat diet alone (HF); high-fat diet supplemented with calafate extract (HFC); high-fat diet supplemented with calafate extract treated with antibiotics (HFCAB), with weight values ranging from 32.8 to 36.9 g. However, there are significant differences with the control mice (C), weighing 28.2 g. In addition, regarding the difference in weights, they observed a minor difference in weights between C and HFCAB compared to HF and HFC; in the four treatments, the values fluctuated between 2.5 and 6.9 g. It should be noted that the weights in the different types of adipose tissues established significant differences in the control mice (C) and the mice with high-fat diets with Calafate extract and antibiotic use (HFCAB); increasing these adipose tissues will affect cardiovascular diseases.

## 6. Potential Bioactivities Demonstrated in Preliminary Studies

The World Health Organization (WHO) reports that cancers are currently among the leading causes of death worldwide [77]. Consuming fruits rich in antioxidants has been linked to a lower risk of developing chronic diseases; however, most available evidence remains limited to experimental or observational studies. In vitro research with calafate extracts has demonstrated a concentration-dependent increase in antioxidant activity, as well as a reduction in the viability of gastric and gallbladder cancer cell lines. These effects were associated with the presence of delphinidin, the main anthocyanidin in calafate, which has been shown to induce apoptosis and decrease cell migration in cancer models [79].

Importantly, these results should be interpreted with caution, as they mainly come from cell-based and animal studies and cannot be directly applied to human clinical outcomes. Some reports also indicate that delphinidin supplementation improves glucose tolerance in rat models with hyperglycemia and exhibits anti-inflammatory activity in vitro [83]. Additionally, Bustamante et al. (2018) [88] suggested that the high antioxidant capacity of calafate berries may be due to their diverse range of anthocyanins. Important antitumor activities against various types of cancer cells have also been reported, with health benefits explicitly attributed to delphinidin-3-glucoside due to its chemical structure [11].

## 7. Conclusions

Calafate (*Berberis buxifolia* Lam.) is a fruit rich in bioactive compounds that contributes to beneficial properties for human health, including antioxidant, anti-inflammatory, and hypoglycemic activities. Furthermore, several studies have indicated a correlation between calafate consumption and a reduced risk of cardiovascular diseases (CVDs). Furthermore, calafate consumption was correlated with substantial modulation of plasma biomarkers associated with cardiovascular disease (CVD), encompassing reductions in thrombomodulin (−24%), sE-selectin (−34%), sICAM-1 (−24%), and proMMP-9 (−31%). Additionally, there was an increase in adiponectin (+68%) and a 17% decrease in plasma hydroxyl radical levels. Notably, it has been suggested that the calafate extract modulates the expression of thermogenic markers, which offers valuable insights into the potential role of this berry in treating obesity-related metabolic diseases.

On the other hand, the lipid content, derived from its seeds, is high in polyunsaturated fatty acids, including α-linolenic acid, linoleic acid, and oleic acid. It also contains tocopherols, a bioactive compound with potent antioxidant and anti-inflammatory properties, positioning it as a potential functional ingredient. Lastly, it has been concluded that calafate may exhibit an antithrombotic function that promotes cardiovascular health, as evidenced by a significant reduction in the Atherogenic Index and the Cardiovascular Risk Index.

The phenolic compound and anthocyanin contents of calafate berries are comparable to those of maqui berries but higher than those of blueberries, positioning them as a valuable fruit in terms of biochemical characteristics.

Limited information and a confined harvest area indicate that calafate berries could be utilized more effectively as fruit. Calafate berries have been used to make jams, beverages, and snacks by hand. Also, although promising preclinical findings exist, the absence of randomized controlled trials (RCTs) in humans remains a significant limitation. Well-structured randomized controlled trials are necessary to determine effective dosages, bioavailability, metabolism, and clinically relevant outcomes, which are crucial for translating Calafate’s bioactive potential into dietary application recommendations.

Future research should expand clinical studies to validate calafate’s health benefits, clarify its molecular mechanisms, and improve extraction and formulation processes for better stability and bioavailability. Additionally, sustainable cultivation and large-scale processing are necessary to maintain quality, availability, and economic viability. These steps are vital for realizing calafate’s potential as a functional food and integrating it into health-promoting diets.

## Figures and Tables

**Table 1:** Proximal analysis of fresh calafate berry.

References Location Country	[13] Punta Arenas Chile	[17] Aysén Region Chile	[27] Patagonia Argentina	
Nutritional Information	Pulp	Pulp	Pulp	Seeds
Energy (kcal/100 g)	98.5	n.m	119.4	144.3
Moisture content (%)	75.9	75.0	93.3	n.m
HC available (%)	14.5	n.m	77.50 *	0.06
Total sugars (%)	9.80	n.m	74.40 *	n.m
Reducing sugars (%)	n.m	n.m	3.10 *	n.m
Proteins (%)	2.80	2.60	1.33	13.65
Fat (%)	0.90	n.m	1.70	18.90
Ash (%)	0.60	0.90	3.65	2.21
Crude fiber (%)	0.10	7.00	n.m	n.m
Total dietary fiber (%)	5.30	n.m	n.m	n.m
Soluble dietary fiber (%)	0.75	n.m	n.m	n.m
Insoluble dietary fiber (%)	4.55	n.m	n.m	n.m

n.m: not measured. * mg equivalents of glucose/g sample.

**Table 4:** Total polyphenol and anthocyanin content and antioxidant activity in calafate berry extracts. Lowercase letters (a–d) indicate significant amplitude differences per ANOVA and Fisher’s test.

References	[74]	[75]	[70]	[76]	[30]	[28]	[39]
Location	Valdivia	Valdivia	Santa Bárbara	Patagonia	Magallanes	Aysén Region	Aysén Region
Country	Chile	Chile	Chile	Argentina	Chile	Chile	Chile
Total polyphenol (mg GAE/100 g d.w.)	1344.2 ± 10.50 ^e^	2233 ± 6.39 ^d^	6553 ± 1.35 ^a^	-	-	2537.5 ± 0.22 ^c^	3600 ± 30 ^b^
Total anthocyanin (mg C-3-G/100 g d.w.)	31.5 ± 0.80 ^d^	66 ± 0.30 ^b^	51.62 ± 1.78 ^c^	-	-	26.5 ± 0.00 ^d^	80.0 ± 5.0 ^a^
Antioxidant activity (mmol Fe^2+^/100 g d.w.)	11.7 ± 1.80 ^b^	38.44 ± 0.08 ^a^	-	-	-	-	-
Antioxidant activity (IC_50_, μg/mL)	-	-	2.33 ± 0.21 ^c^	12.6 ± 0.38 ^b^	-	-	25.0 ± 1.0 ^a^
Antioxidant activity (μmol TE/g d.w.)	-	-	124.46 ± 6.54 ^a^	-	62.75 ± 3.10 ^b^	-	63.0 ± 5.0 ^b^

**Table 5:** The primary findings that underscore the biological activities of calafate (*Berberis buxifolia* Lam).

Biological Activity	Preparation/Extract/Compound	Experimental Model	Major Outcomes	Reference
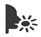 Anti- inflammatory	Polyphenol-rich berry extract	In vitro (macrophages stimulated with LPS); rodent models of diet-induced obesity	↓ Pro-inflammatory markers; ↓ NO secretion; modulation of cytokine expression; ↓ fat deposits; improvement of insulin sensitivity	[74,75,83]
	Leaves and roots (alkaloid fraction)	In vitro (antibiotic activity assays)	Reported antibiotic and anti-inflammatory properties; limited vasodilator evidence.	[84]
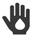 Hypoglycemic	Ethanolic root extract	Hepatic cell culture	↑ Glucose uptake correlated with dose	[15]
	Berry extract (polyphenol-rich)	In vivo (diet-induced obesity, mice)	Restoration of glucose tolerance; improvement of insulin resistance	[75]
	Fruit extract	In vitro (adipocytes under insulin-resistant conditions)	Inhibition of glucose uptake by adipocytes	[83]
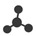 Antioxidant	Fruit extract	In vitro assays (DPPH, ABTS, FRAP, ORAC); human erythrocytes (lipid peroxidation assay)	↑ Radical scavenging activity; protection against lipid peroxidation; antiradical capacity correlated with phenolic and anthocyanin content	[17,70,71]
	Pulp, peel, seeds (organ-specific)	Analytical assays	Pulp/peel: anthocyanins and hydrophilic phenolics; seeds: lipids and tocopherols; organ-dependent antioxidant roles	[20]
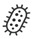 Antimicrobial	Roots, stems, leaves (methanol/ethanol extracts, alkaloid fraction rich in berberine)	In vitro (Gram-positive bacteria: *S. aureus*, *B. cereus*, *S. epidermidis*, *B. subtilis*)	Inhibition of bacterial growth; activity linked to isoquinoline alkaloids (mainly berberine)	[9,11,85]
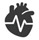 CVD risk-reducing/Metabolic modulation	Polyphenol-rich berry extract	In vivo (mice, chronic administration)	↓ Plasma biomarkers (thrombomodulin, sE-selectin, sICAM-1, proMMP-9); ↑ adiponectin; ↓ OH radicals; protective effects against atherosclerosis	[7]
	Freeze-dried calafate	In vivo (mice)	↓ Atherogenic Index and CVD Risk Index; no significant effect on cholesterol or triglycerides	[86]
	Acute and chronic berry extract administration	In vivo (mice)	Modulation of thermogenic markers; prevention of mitochondrial and microbiota alterations in obesity models	[8]

↓ decrease; ↑ increase.

## Data Availability

The original contributions presented in the study are included in the article; further inquiries can be directed to the corresponding author.
